# Behavioral predictors of subsequent respiratory illness signs in dogs admitted to an animal shelter

**DOI:** 10.1371/journal.pone.0224252

**Published:** 2019-10-23

**Authors:** Alexandra Protopopova, Nathaniel J. Hall, Kelsea M. Brown, Allison S. Andrukonis, Jessica P. Hekman

**Affiliations:** 1 The University of British Columbia, Faculty of Land and Food Systems, Vancouver, British Columbia, Canada; 2 Texas Tech University, Department of Animal and Food Sciences, Lubbock, Texas, United States of America; 3 The Broad Institute of MIT and Harvard, Vertebrate Genomics Group, Boston, Massachusetts, United States of America; Universidade do Porto Instituto de Biologia Molecular e Celular, PORTUGAL

## Abstract

Individual variability is evident in behavior and physiology of animals. Determining whether behavior at intake may predict subsequent illness in the animal shelter may influence the management of dogs housed at animal shelters and reduce overall disease. While normally associated with mild disease and low mortality rates, respiratory disease nevertheless poses significant challenges to the management of dogs in the stressful environment of animal shelters due to its highly infectious nature. Therefore, the aim of the study was to explore whether behavior at intake can predict subsequent occurrence and progression of upper respiratory disease in dogs at animal shelters. In a correlational study, 84 dogs were assessed throughout their stay at a city animal shelter. The dogs were subjected to a behavioral assessment, 1 min in-kennel behavioral observations across two observation periods, and the collection of urinary cortisol:creatinine (C:C) ratio. The occurrence and progression of upper respiratory disease was monitored through repeated clinical exams (rectal temperature and the occurrence of nasal and ocular discharge, and presence of coughing and sneezing). A basic PLS Path regression model revealed that time in the shelter (estimate = .53, p < .001), and sociability (estimate = .24, p < .001) and curiosity scores (estimate = .09, p = .026) were associated with increased illness. Activity and anxiety scores, however, were not associated with illness. Urinary C:C, taken on the first full day, did not predict subsequent illness when accounting for time. Limitations included attrition of dogs, a small percentage receiving vaccinations, and continuous and non-systematic rotation of dogs in the kennels. Understanding if behavior can predict subsequent illness may improve shelter management practices, and in turn, result in improved live-release outcomes.

## Introduction

Respiratory disease, while normally associated with mild symptoms and low mortality rates, poses significant challenges to the management of dogs in the highly stressful environment of animal shelters due to its highly infectious nature[1]. Canine infectious respiratory disease complex (CIRDC), also known as kennel cough complex, is composed both of bacterial and viral agents associated with low mortality but high prevalence (canine adenovirus-2, canine parainfluenza virus, canine respiratory coronavirus, canine herpesvirus, canine influenza, *Mycoplasma bronchiseptica*, and *Mycoplasma cynos*) and agents associated with high mortality though much lower prevalence (canine distemper virus and *Streptococcus equi* subsp. z*ooepidemicus*[1–3]). CIRDC signs include ocular and nasal discharge and cough[1]. The highly infectious nature of the complex has implications for animal shelters due to the need to isolate symptomatic animals, inability to neuter infected animals, and the reluctance of potential adopters to bring infectious animals into their homes, where they may have other dogs. Associated veterinary care for symptomatic animals also taxes limited shelter resources[4]. Finally, canine distemper and Strep zoo, though less prevalent than the others in the complex, can be associated with significant loss of life in a crowded shelter[5]. The outcome of these challenges is that many shelter dogs exhibiting signs of CIRDC may be euthanized rather than placed for adoption if there are budget and/or time constraints[6,7].

Not only does the shelter provide an ideal setting for disease transmission, with dogs housed in close proximity to each other, but the stress of the shelter environment likely reduces the immune system's ability to respond to microbial challenge[1,8]. Differential susceptibility to various pathogens has been well established in young dogs and pregnant females[9] as well as individuals who are immunocompromised due to an already established disease (e.g., feline immunodeficiency virus[10]). However, other less obvious factors, such as increased stress, may increase susceptibility in the shelter. The shelter environment presents an array of psychosocial stressors for dogs, resulting in increased activity of the hypothalamic-pituitary-adrenal (HPA) axis, as indicated by elevated cortisol levels in animals in that environment compared to animals in pet homes, at least in the first few days after admittance[11]. The HPA axis is the pathway that is responsible for the activation of the stress response in animals. An environmental stressor triggers the hypothalamus to release corticotropin-releasing hormone (CRH) and arginine vasopressin, which, in turn, stimulate the production of adrenocorticotropin hormone (ACTH) in the anterior pituitary. The release of the latter hormone stimulates the release of corticosterone or cortisol from the adrenal cortex into the blood stream, depending on the species[12,13]. The high levels of cortisol (or corticosterone) inhibit further production of the CRH and ACTH resulting in a negative feedback loop (see [14] for a discussion in shelter dogs). Cortisol levels negatively correlate with the levels of secretory immunoglobulin A (s-IgA) in dogs[15]. Yet s-IgA plays a central role in the mucosal immune system, the body’s primary defense against infection of the respiratory system[16].

Individual variability is evident in behavior and physiology of human and non-human animals; individuals tend to cope with stressors in systematic and consistent ways[17]. Correlations between behavior and physiological ability to cope with environmental stressors, such as disease and parasite infection, have been demonstrated in a wide variety of species. Capitanio, Medoza, and Baroncelli[18] found that rhesus macaques that were high in the sociability trait showed a more rapid decrease in plasma cortisol concentrations, a higher IgG response, and a lower viral load when challenged with a simian immunodeficiency virus. Pigs that spent more time struggling when flipped over on their backs for a brief amount of time, have been found to have a higher concentration of cortisol and lower immune function[19]. Free-roaming tom cats that were higher in aggression had a higher viral load of feline immunodeficiency virus[20]. Trapped Norway rats, who had a higher presence of wounds (as an indicator of aggression), also showed a higher level of hantavirus infection, and in turn, higher infection status males showed elevated serum testosterone and corticosterone concentrations, among other differences in neurotransmitters[21]. The activity-exploration profiles of Siberian chipmunks, as measured by a standardized test, predicted the numbers of ticks present on the animals; tick load increased with space use[22]. More recently, the predictive effect of behavior on immune function under chronic stress conditions has been extensively explored in cattle[23,24]. Temperamental cattle, those that display shorter latency to exit and higher velocity when exiting their enclosure, have been shown to have a higher cortisol concentration and a weaker immune response to pathogens[23].

The predictive nature of behavior on immune function or disease status in dogs has not received much attention. However, early prediction of illness in a shelter environment may lead to higher life-saving through improvements in population management. To decrease overall disease within an animal shelter, experts recommend removing sick animals from the population as well as differential treatment of those that are more likely to succumb to disease[25]. Thus, characterizing dogs on intake into high and low- risk categories may decrease overall incidence of disease in animal shelters as well as protect those that are more susceptible.

Recently, Corsetti et al.[26] have suggested that dogs displaying a bold personality were less susceptible to diseases in the animal shelter. The researchers assessed 28 dogs for one month at the shelter. The behavior of the dogs was assessed using standardized personality tests, including a T-maze and a novel object test. The complex “boldness” trait was statistically derived from scores from several other tested traits (e.g., activity, attentiveness, dominance, and sociability). The aim of the study was to extend the work of Corsetti et al. by exploring whether behavior at intake can predict the subsequent development of CIRDC in dogs surrendered to animal shelters.

## Materials and methods

### Animals and housing

Adult dogs of unknown breed (n = 84; those that appeared to be approximately 1 year of age and older; 61% male) housed at the Lubbock Animal Shelter, a city shelter in Lubbock, Texas, were enrolled in the study from February through November, 2016. The shelter is an open-admission shelter, which admits owner-surrendered, stray, and confiscated animals. The dogs which are available for adoption are placed into a separate kennel area, with the remaining dogs in a different area. For this study, only dogs that were not in the adoptable area were enrolled. Dogs were excluded from the study due to the presence of sign of illness (e.g., nasal discharge, coughing, etc.) on Day 1, pronounced aggression towards the experimenters, obvious injury, and mothers with litters.

Dogs were group housed, with some exceptions, in 1.6m x 1.2m x 1.9m steel kennels with cement siding and floors. A guillotine door divided the two parts of each kennel. Occasionally the guillotine door was raised and the dogs were given access to both of the kennels. Dogs who were dog-aggressive, as evidenced by fighting when group housed, were placed into the kennel alone. The kennels contained a water and food bowl. Staff cleaned the kennels and fed the dogs daily. Dogs were not routinely taken out of their kennels. Occasionally the kennels had a towel or blanket in them. The front two rows of kennels were for male dogs and the middle two rows were for female dogs. The last row of kennels was used for aggressive, pregnant/lactating or injured dogs. There were drainage grates directly in front of the kennels and a cement walkway in between rows of kennels.

Eleven dogs (13%) received vaccinations (combined canine distemper virus, hepatitis, parvovirus, and parainfluenza administered subcutaneously, and Bordetella administered intranasally) after intake (one dog each on Days 1 and 2, two on Day 3, one each on Days 6, 7, 8, 9, and 10, and two on Day 11). However, no systematic programs to vaccinate dogs on intake were present at the time of the study in the shelter, with an approximate >80% unvaccinated (at intake) dogs present at the shelter during the study. The new dogs were placed into kennels with existing unvaccinated dogs, thus, it is very likely that the animals were already exposed to pathogens prior to developing immunity even for the few dogs that were vaccinated on Day 1. Moreover, efficacy of vaccination against CIRDC is variable, and the most significant predictive factor in whether a dog contracts CIRDC may be the number of days in the shelter, rather than vaccination status[5,27].

### Ethical considerations

While the housing practices, husbandry, and outcome decisions were beyond our control and strictly at the shelter staff’s discretion, our study procedures were approved by the Texas Tech University Institutional Animal Care and Use Committee (#15064–09). Dogs were handled gently, with care and respect, and we tried to be the best part of their otherwise stressful day. Withholding vaccination from all dogs would have made our results easier to interpret. However, dogs are routinely vaccinated in shelters with multivalent vaccines that include highly effective protection against lethal diseases such as distemper virus and parvovirus. For this reason, withholding vaccination for the purposes of this study was not deemed ethical. At the time of the study, the animal shelter had poor disease management practices, including poor sanitation, poor medical care, no vaccination at-intake, overcrowding, and continuous rotation of dogs in the kennels. Since the time of our study, welfare improvements have been made, including intake vaccinations, improved medical care and sanitation practices.

### Data collection

Dogs were enrolled in the study the day after they arrived at the shelter (arrival day was coded as Day 0, and data collection began on Day 1). The dogs’ intake ID number, intake date, and kennel tag number were recorded. There were two cohorts of dogs: those for whom data collection occurred on Monday, Wednesday, Friday and those for whom data collection occurred on Tuesday, Thursday, and Saturday. On Day 1 to Day 14 (a total of seven observations), the dogs were videotaped in their kennel for 1 min using a Kodak PIXPRO SPZ1, while one or two experimenters stood passively in front of the kennel. The behaviors during the in-kennel videos were coded at a later time. This short observation period was previously used to detect behavioral differences across kenneled dogs in the animal shelter environment[28,29]. On Days 1 through 14, using a slip lead, the dogs were led outside into a 34m x 26m fenced yard and the dog’s health was assessed. The yard contained synthetic grass and concrete with a large window looking into the yard from inside the shelter. On Day 3, prior to the health assessment, a behavioral assessment was conducted and saliva and urine were collected. If insufficient volumes of these samples were obtained, the sample collection was repeated for Day 5 or 6. Saliva samples were collected prior to the health exam using inert polymer swabs (SalivaBio Children’s Swab, Salimetrics, Carlsbad, CA, USA) held in the dog’s mouth for 60 s, but were lost due to human error during analysis; therefore, no data are reported.

#### Health observation

At least two researchers were present to conduct the health assessment. The presence of coughing and sneezing, and nasal and ocular discharge were noted during approximately 1 min observations of the dog in the kennel (these signs were noted as important through conversations with several experienced shelter veterinarians). The operational definitions of these categories are listed in Table 1. The dog’s body condition score (Purina Body Condition System[30]) with a range of 1–9 was recorded. On few occasions, the dog was too sick or distraught to exit their kennel; in that case, the health assessment occurred inside the kennel.

**Table 1:** Operational definitions of categories during the health observations.

(Score) Description	Operational Definition	
**Ocular discharge**	**(0) No discharge**	Eyes appear clear, not swollen or red, with no discharge
	**(1) Clear discharge**	Discharge is transparent in color. Eyes appear watery
	**(2) Swollen**	The eyeball or the eyelids appear swollen. Eye may or may not be red
	**(3) Yellow/green discharge**	Yellow or green, opaque, and viscous discharge present
**Nasal discharge**	**(0) No discharge**	Nose is relatively dry with no crusting
	**(1) Clear discharge**	Nose appears wet, transparent drops of discharge evident
	**(2) Yellow/ green discharge**	Opaque and viscous yellow or green colored discharge
	**(3) Crust on nose**	Crusting on and around the nose is present
	**(4) Bloody discharge**	Red colored discharge is present
**Coughing** **(0) Absent** **(1) Present**	Sudden, noisy, forceful expulsion of air from the lungs while the mouth remains open. May sound similar to human hacking	
**Sneezing** **(0) Absent** **(1) Present**	Sudden, noisy, forceful expulsion of air from the lungs through the nose (the mouth likely to be closed). Sounds similar to human sneezing	

While one experimenter briefly and gently restrained the dog (when necessary), the second obtained a rectal temperature twice. If the two values differed by more than 0.1°C, the temperature was taken a third time. The dogs were fed dog treats (Pup-Peroni® Dog Snacks, Big Heart Pet Inc., San Francisco, CA, USA) throughout the health assessment as a distraction. Dogs that consistently refused to allow for the collection of rectal data were excluded from further procedures and data analysis; three dogs refused several times during their stay, resulting in ten missing time points (out of 403 time points total).

On 22.6% of observations, the two observers collected data independently in order to calculate inter-observer agreement. Cohen’s kappa (κ) was calculated to determine agreement between the two observers in their score determination for the condition of the nose, the eyes, and the presence of coughing and sneezing. There was high agreement for all four measures, κ_nose condition_ = .84 (95% CI, .78 to .89), κ_eye condition_ = .73 (95% CI, .63 to .80), κ_coughing_ = .88 (95% CI, .65 to 1), κ_sneezing_ = .86 (95% CI, .75 to .98).

#### Behavioral assessment

Following the collection of 1 min video clips of in-kennel behavior, the videos were coded on all behaviors listed in Table 2. Videos were coded using a partial-interval coding procedure with 5 s time bins, in which an occurrence or non-occurrence of each behavior was noted. The behavioral dependent variables were derived by taking the proportion of the time bins in which a behavior occurred. A portion of the videos (24%), selected at random, were double coded. Inter-observer agreement was calculated by adding the number of agreements by interval, dividing by the total number of intervals, and multiplying by 100. The inter-observer agreement was calculated for each behavior independently by summing all agreements of whether or not a behavior occurred in that interval, dividing by the sum of agreements and disagreements, and multiplying by 100. The average agreement across behaviors was 99% (SD = 0.01%; min: 95% for “leaning on wall,” max: 100% for “out of sight,” “chasing tail,” “lying down,” “cowering,” “tucking tail,” “growling,” “howling,” and “leg lift”).

**Table 2:** Operational definitions of all of the behaviors that were observed during the in-kennel observation period.

Behavior	Operational Definition
**Front of kennel**	Located between front of cage, and up to and including the midpoint of the visible kennel
**Back of kennel**	Located between back wall of kennel, and up to, but not including, midpoint of the visible kennel.
**Out of sight**	Not visible from the front of the cage, behavior cannot be defined
**Facing forward**	Head is oriented such that the observer is able to see more than the side profile of face
**Gazing**	Likely eye contact with the eyes of the observer
**Facing away**	Head is oriented such that the observer is not able to see more than the side profile of face
**Moving forward**	Distance between the dog and the observer is decreased
**Moving away**	Distance between the dog and the observer is increased
**Jumping on cage**	Both front paws make contact with the cage door that does not include lunging
**Chasing tail**	Orients towards tail repeatedly (more than 3 times) and continuously
**Pacing**	Repeatedly (more than 3 steps) locomoting around kennel in fixed route
**Standing**	Supported upright with all four legs
**Sitting**	Supported by two extended front legs and two flexed back legs
**Lying down**	Lying down with limbs either tucked under or placed in front of body
**Pawing at door**	One front paw makes contact with the cage door
**Cowering**	Body in a lowered, crouched position
**Wagging tail**	Tail moves perpendicular to the dog’s body
**Tucking tail**	Tail held still and tightly between hind legs, may be curled under genital area or ventral side
**Barking**	Vocalization of very short duration and low frequency
**Whining**	A cyclic vocalization
**Growling**	Throaty, rumbling vocalization; usually low in pitch
**Howling**	Prolonged high-amplitude vocalization of varying pitch, lips drawn together while exhaling
**Leaning on wall**	Prolonged (more than 1sec) contact with the cage wall by pushing side of body against the cage wall
**Leaning on door**	Prolonged (more than 1 sec) contact with the cage door by pushing side of body against the cage door
**Licking cage**	Repeatedly chews, licks, and/or bites at the wire of the cage door
**Licking self**	Oral contact with any part of body
**Shaking off**	Motions body and/or head back and forth repeatedly and rapidly
**Scratching**	Paw makes repeated contact with body/face; head may be angled in direction of moving limb
**Yawning**	Opens mouth widely and inhales
**Stretching**	Extending body and one or more front and/or hind-legs while remaining stationary
**Panting**	Tongue exposed with audible and/or observable breathing
**Trembling**	Visible shaking while dog is standing still or cowering
**Leg lift**	Any leg is lifted off the ground for 3 sec or more

The behavioral assessment was modified from Hennessy et al.[31] to contain three components to measure Activity, Sociability, and Boldness/Curiosity. A 1m x 1m square was marked off using adhesive measuring tape in the outdoor yard. The dogs were first allowed a few minutes to habituate to the area as well as to toilet (urine was collected at this time; see below for details). The first component of the test consisted of the researcher allowing the dog to remain alone, unrestrained, in the outdoor yard. The researcher videotaped the dog through the window for 2 min. The second component of the test included the researcher kneeling in the center of the 1m x 1m square for 3 min. If the dog had two or more paws inside the square, the researcher would pet and praise the dog using the hand closest to the body of the researcher to allow for the dog to escape at any moment. A second researcher videotaped the interaction through the window. The third part of the test involved the placement of a clear plastic tub, with a remote-control car inside, placed within a 1m x 1m square on the side of the play yard. One researcher stood approximately 1.5 m away from the car and controlled it using a remote to continuously and erratically drive the car inside the tub. The other researchers stood on the opposite side of the pen and videotaped the interaction for 2 min.

These videos were coded on the behaviors listed in Table 3 and additionally on the corresponding behaviors listed in Table 4. The videos were coded using the partial-interval coding procedure with 5 s time bins, in which an occurrence or non-occurrence of the behavior was noted. The behavioral variables were derived by taking the proportion of the time bins in which a behavior occurred. A portion of the videos (29%), selected at random, were double coded. Inter-observer agreement was calculated by adding the number of agreements by interval, dividing by the total number of intervals, and multiplying by 100. Inter-observer agreement was calculated for each behavior independently. The average agreement across behaviors was 99% (SD = 0.01%; min: 97.3% for “walking,” max: 100% for “jumping on fence,” “howling,” “tail tucked,” “cowering,” “cowering,” “body trembling,” “grab car,” and “play bow”).

**Table 3:** Operational definitions of the behaviors that were observed in all three components during the behavioral assessment.

Behavior	Operational Definition
**Standing**	Supported upright with all four legs
**Sitting**	Supported by two extended front legs and two flexed back legs
**Lying down**	Lying down with limbs either tucked under or placed in front of body
**Walking**	Dog is walking (4-gait movement)
**Trotting**	Dog is trotting (2-gait movement)
**Galloping**	Dog is galloping (3-gait movement)
**Near door frame**	Any part of dog is arm's length (1m) or closer away from door
**Leaning on fence**	Any art of the body in contact with fence or wall
**Jump on fence**	At least one paw is on wall or fence
**Jump on glass**	At least one paw is on glass wall
**Barking**	Vocalization of very short duration and low frequency
**Whining**	A cyclic vocalization
**Howling**	Prolonged high-amplitude vocalization of varying pitch, lips drawn together while exhaling
**Tail tucked**	Tail is between or further the hind legs
**Cowering**	Body in a lowered, crouched position

**Table 4:** Operational definitions of the additional behaviors that were observed in the sociability and the curiosity components during the behavioral assessment.

Behavior		Operational Definition
**Sociability**		
	**Proximity to person**	At least two paws are within or on the tape measuring out 1m^2^ around the person
	**Jump on person**	At least one paw placed on the person
	**Lean on person**	Body contact with the person (excluding just the person's hand)
**Curiosity**		
	**Gazing at car**	The eyes of the dog are directed at the car
	**Approach car**	Distance between the dog and the car is decreased and gaze directed at car
	**Retreat from car**	Distance between the dog and the car is increased. "Approach car" had to have happened right before or dog was in "Proximity to car" right before
	**Proximity to car**	At least one paw is within or on the tape measuring out 1m^2^ around the car
	**Grab car**	Dog grabs the car with mouth

#### Urinary cortisol:Creatinine ratio

The urine was collected using a clean plastic vial. Immediately following collection, urine was labeled and placed in a cooler with ice packs. Following the collection of the urine for the day, the samples were taken to a secured freezer in the Texas Tech University Animal and Food Sciences Building. Twenty dogs did not urinate when taken outside, resulting in 64 of dogs containing urinary C:C data.

The urine was shipped, in dry ice, to an independent Texas A&M Veterinary Medical Diagnostic Laboratory (College Station, TX, USA) for analysis. A cortisol radioimmunoassay from MP Biomedicals (MP Biomedicals, LLC, Santa Ana, CA, USA) was utilized. Urine was extracted with dichloromethane using 0.5 ml of urine with 1 ml of solvent. The procedure involved first mixing for 5 min followed by 5 min of rest. Following rest, the sample was dried down under nitrogen (50 μl per tube) and 25 μl of stripped canine serum was used to the quantification standard of the assay kit (25 μl of standard, control, and target serum).

### Data analysis

#### Health coding

The health observations (Table 1) were coded to provide a quantifiable severity of the observation as an “illness score”. The coding scheme for translating observations to a numerical score is presented in the same table. The median rectal temperature was coded as a numerical value.

#### Behavior coding and filtering

When considering each behavioral variable for each observation period, 254 behavioral variables were scored across the study period. We implemented a variable quality selection procedure. First, we removed all variables in which fewer than 8 dogs (~10% of all dogs) exhibited across the study period. This filter retained 201 variables. Second, we removed all variables that showed little variability across dogs. Variables with a standard deviation of less than .01 were removed, leaving a total of 141 behaviors. Because the in-kennel behaviors were evaluated at multiple time points, we further restricted our analysis to use the behavioral variables from the first two observation days to predict health observations across the 14 study days. All data are available in the supplementary materials; however, for the aims of this study to predict health outcomes, we restricted our analysis on the *first two* observation periods (Day 1 and Day 3). Only 56 behavioral variables remained for exploration.

#### Path analysis

To identify whether temperament influenced overall health, we conducted a path analysis using the *plspm* package in R[32]. To test the hypothesis that Curiosity, Sociability, Anxiety, and Activity may impact illness risk, the coded behaviors were categorized into latent variables by the first author based on previous research by Hennessy et al. (see Table 5[31,33–36]). Each behavioral indicator variable was included as a reflective indicator of the latent variable. For the classification of behaviors not listed in the previous study, we attempted to group similar behaviors into established categories. For example, “gazing” and “proximity” to car were grouped with “approach” to car. In-kennel behaviors were interpreted taking into account previous research that showed that some behaviors correlated strongly together and were emitted by dogs when a person was actively interacting with them through the kennel[29]. Previous research has shown that “back of kennel” is highly correlated with “front of kennel” and can be considered in unison; the same phenomenon occurs for “facing forward” and “facing backwards”. Combined, these can be labeled “back and forth facing or motion” as was done in Protopopova et al. An example of this phenomenon can be demonstrated by observing a dog pacing back and forth in the kennel. Because “gazing”, “jumping on cage”, “barking”, “whining”, and “wagging tail” increased in previous research when a person actively solicited attention, these may be considered as part of sociability[29]. Hekman and colleagues previously found that panting and lip licking were positively correlated with salivary cortisol concentrations, indicating stress[37]. Thus, we included “panting” and “licking self” into the “anxiety” latent variable. “Leaning on the wall” has previously been found to correlate with a long length of stay at the shelter[29], which may indicate some form of distress. Therefore, we elected to place this behavior into the “anxiety” latent variable; however, we recognize that this was a subjective decision. “Barking” and “proximity to the experimenter” during the boldness/shyness test were logically grouped into the “anxiety” latent variable as they may have indicated distress of the dog as a result of the toy car; again, we recognize that this was a subjective decision. All activity-related behaviors were grouped into the “activity” latent variable.

**Table 5:** Indicator variables for each temperament latent construct. Four latent variables (Curiosity, Sociability, Anxiety, and Activity) were indicated by four or more behavioral variables. Every variable name begins with the context of the behavior (Bold/shy test [BoldShy], sociability test [Sociability], activity test [Activity], and in-kennel behavior observations on Day 1 [Kennel1] and Day 3 [Kennel2]).

Curiosity	Sociability	Anxiety	Activity
BoldShy.ApproachCar ([31], “wariness”)	Kennel1.Back_of_kennel[29]	BoldShy.Barking	Kennel1.Lying_down
BoldShy.GazingAtCar	Kennel2.Back_of_kennel[29]	Activity.JumpOnGlass ([31], “timidity/flight”)	Kennel2.Lying_down
BoldShy.ProximityCar	Kennel2.Barking ([31], “solicitation”)	Kennel1.Leaning_wall[29]	Sociability.LyingDown
BoldShy.RetreatCar ([31], “wariness”)	Kennel1.Facing_away[29]	Kennel2.Leaning_wall[29]	BoldShy.LyingDown
	Kennel2.Facing_away[29]	Kennel1.Licking_self[37]	Activity.Sitting
	Kennel1.Facing_forward[29]	Kennel2.Licking_self[37]	BoldShy.Sitting
	Kennel2.Facing_forward[29]	Activity.NearDoorFrame ([31], “timidity/flight”)	Kennel1.Sitting
	Kennel1.Front_of_kennel[29]	BoldShy.NearDoorFrame ([31], “timidity/flight”)	Kennel2.Sitting
	Kennel2.Front_of_kennel[29]	Sociability.NearDoorFrame ([31], “timidity/flight”)	Sociability.Sitting
	Kennel1.Gazing[29]	Kennel1.Panting[37]	Activity.Standing
	Kennel2.Gazing[29]	Kennel2.Panting[37]	BoldShy.Standing
	Kennel1.Jumping_on_cage ([31], “solicitation”)	BoldShy.ProximityPerson	Kennel1.Standing
	Kennel2.Jumping_on_cage ([31], “solicitation”)		Kennel2.Standing
	Sociability.JumpOnPerson ([31], “sociability”)		Sociability.Standing
	Sociability.LeanOnPerson ([31], “sociability”)		Activity.Trotting
	Sociability.ProximityPers ([31], “sociability”)		BoldShy.Trotting
	Kennel1.Wagging_tail[29]		Activity.Walking
	Kennel2.Wagging_tail[29]		BoldShy.Walking
	Kennel1.Whining ([31], “solicitation”)		Sociability.Walking
	Kennel2.Whining ([31], “solicitation”)		
	BoldShy.Whining ([31], “solicitation”)		

An initial model was fit and the loadings, cross loadings, Cronbach's alpha, and Dillon-Goldstein's rho were checked to evaluate the unidimensionality of the temperament and health variables. Indicator variables that were poorly correlated with their respective latent variable (Cronbach’s alpha & Dillon-Goldstein’s Rho < .4) were removed from consideration. The model was re-evaluated and temperament and health variables were assessed for unidimensionality with a raised criterion of .5 for Cronbach’s alpha and Dillon-Goldstein’s Rho. The remaining indicator variables were deemed acceptable for inclusion.

**Fig 1:**
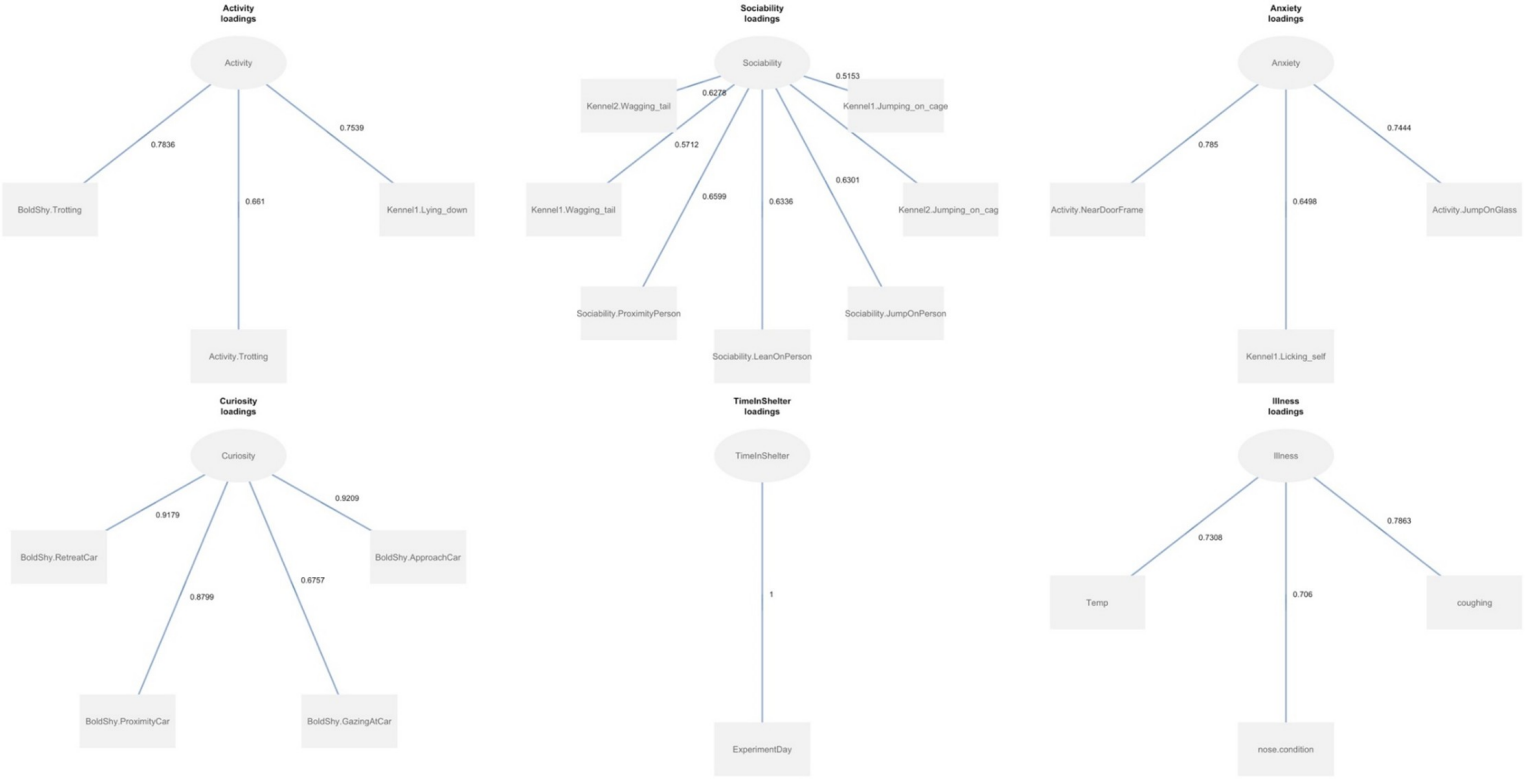
Loadings of all of the variables in the final model. Raw data as well as all codes from the statistical software R are available as supplementary material (S1 File).

To evaluate whether the latent temperament variables were associated with health, we proposed a basic structural model in which Curiosity, Sociability, Anxiety, Activity, and Time in the Shelter independently predicted Illness (Fig 1). Details of this model are described in the results.

## Results

### Descriptive data

**Fig 2:**
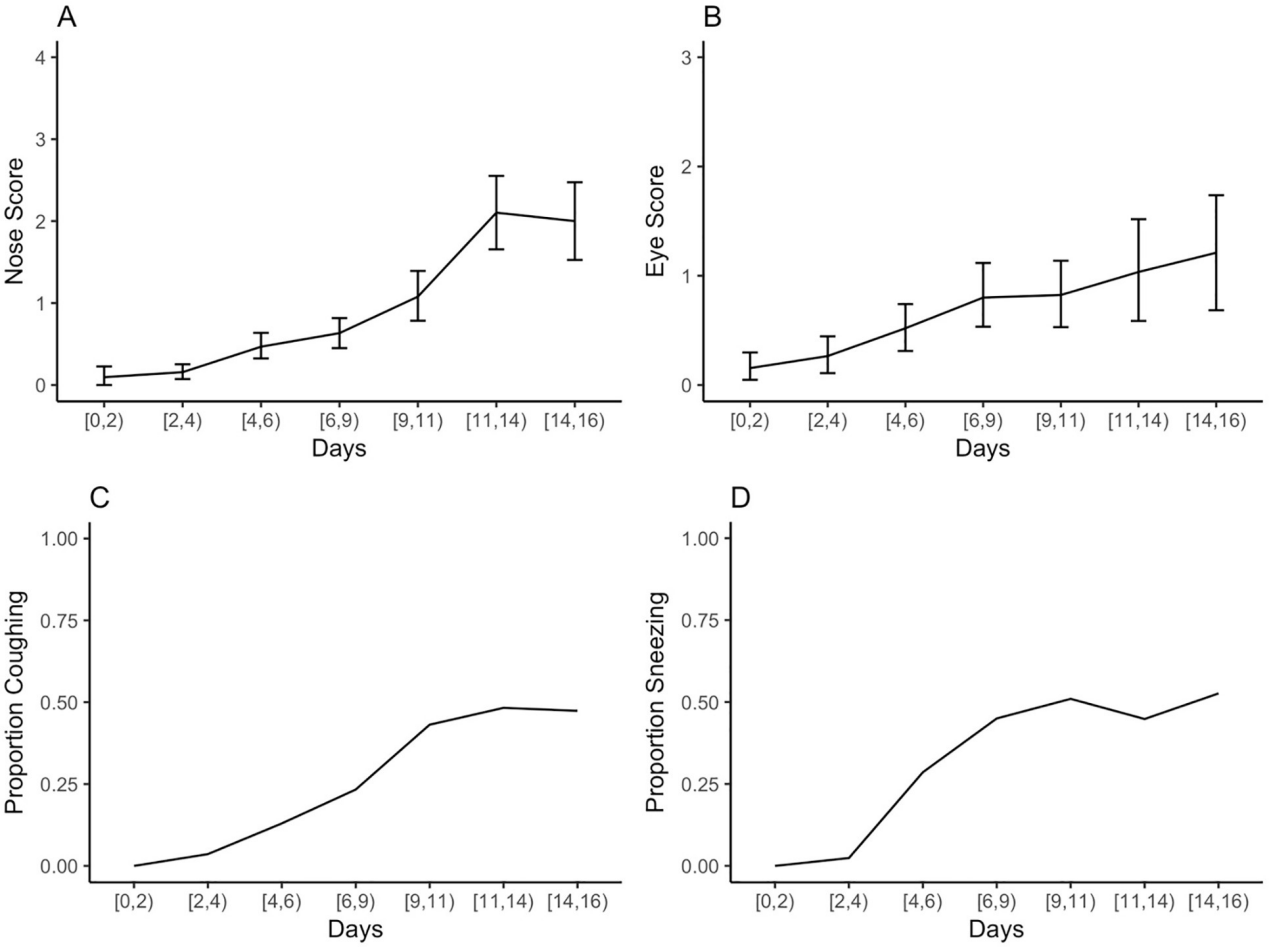
The nose and eye condition and coughing as a factor of days spent in the shelter. A) The mean nose condition (range: 0–4; 0—no discharge, 1—clear discharge, 2—colored discharge, 3—crust, 4- bloody discharge) as a factor of days spent in the shelter. Error bars represent 95% confidence intervals. B) The mean eye condition (range: 0–3; 0—no discharge, 1—clear discharge but no irritation, 2—clear discharge with irritation, 3—pus) as a factor of days spent in the shelter. Error bars represent 95% confidence intervals. C) The proportion of coughing as a factor of days spent in the shelter. D) The proportion of sneezing as a factor of days spent in the shelter. Note that the number of dogs were decreasing across days. See attrition rates in Table 6.

**Fig 3:**
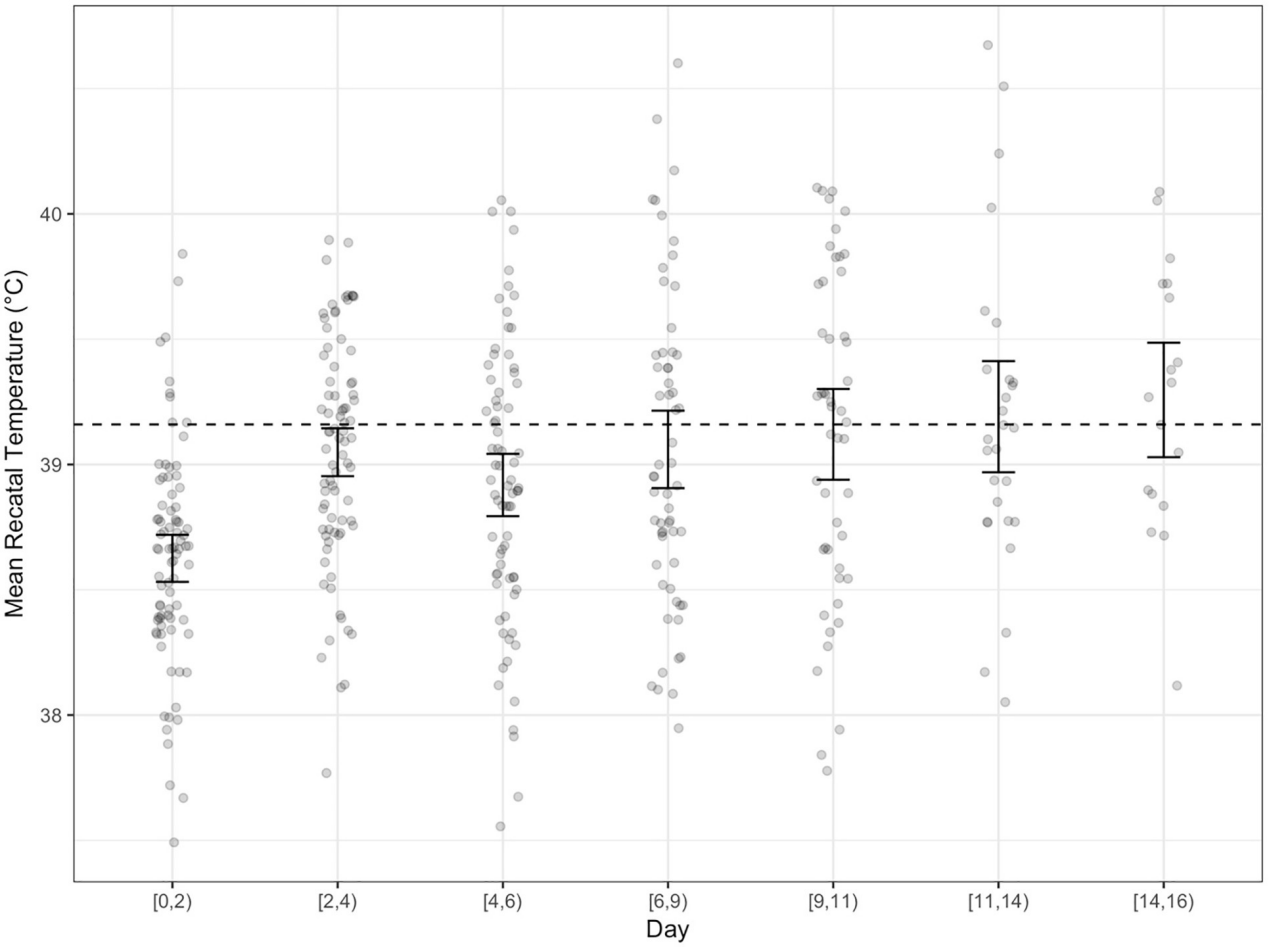
The mean rectal temperature (in celsius; C) as a factor of days spent in the shelter. Error bars represent 95% confidence intervals and dots represent individual data. A horizontal line at 39.16°C represents a determination of fever.

Table 6 shows the attrition from the study. Dogs were euthanized (83%), sent to the adoption floor (7%), returned to owner (6%), or died in their kennel (3.5%). Most dogs were available for the behavioral assessments through the first 3–4 days; however, only 19 dogs remained by day 14. During this time, health became progressively worse. Fig 2 shows the change in the health observations across time. Health steadily worsened as indicated by increases in the severity of the illness observation scores. In addition, temperature increased indicative of fever. Fig 3 shows the mean rectal temperature across time as well as the 95% confidence interval (boot-strapped confidence intervals obtained via packages ggplot2)[38]. The dotted line indicates the threshold for fever (39°C). At study initiation, the 95% confidence interval was well below the fever threshold. However, by Day 8 through the end of the study, the 95% confidence interval overlapped with a fever threshold. Together, these results clearly indicate the development of illness and systematic increase in severity across the study period.

**Table 6:** Attrition of dogs from the study across time.

Days	Number of dogs remaining in study
1	84
3–4	83
5–6	77
7–9	60
10–11	51
12–14	29
15	19

### Path model results

Through our established exclusion criterion for relatedness of a behavioral variable to the latent variable, 32 variables were with Cronbach’s alpha and Dillon-Goldstein’s Rho < .4. In a final step, 21 variables with a Cronbach’s alpha and Dillon-Goldstein’s Rho ≥ .5 were retained in the final model. The final Cronbach’s alpha and Dillon-Goldstein’s Rho for each retained variable are shown in Table 7 and Table 8 shows latent variable loadings.

**Table 7:** Latent variable unidimensionality.

	Cronbach’s alpha	Dillon-Goldstein’s Rho
**Activity**	0.58	0.78
**Sociability**	0.74	0.82
**Anxiety**	0.56	0.77
**Curiosity**	0.87	0.92
**Time in Shelter**	1.00	1.00
**Health**	0.59	0.79

**Table 8:** Latent variable loadings.

Behavior	Latent Variable	Activity	Sociability	Anxiety	Curiosity	TimeInShelter	Illness
Kennel1.Lying_down	Activity	0.75	0.29	0.28	0.25	-0.02	0.12
Activity.Trotting	Activity	0.66	0.16	0.17	0.05	0.04	0.07
BoldShy.Trotting	Activity	0.78	0.26	0.31	0.17	0.03	0.11
Kennel1.Jumping_on_cage	Sociability	0.32	0.52	0.33	0.13	0.09	0.17
Kennel2.Jumping_on_cage	Sociability	0.20	0.72	0.30	0.43	0.08	0.25
Sociability.JumpOnPerson	Sociability	0.05	0.63	0.42	0.24	0.03	0.31
Sociability.LeanOnPerson	Sociability	0.23	0.63	0.30	0.25	0.04	0.16
Sociability.ProximityPerson	Sociability	0.17	0.66	0.22	0.22	0.07	0.23
Kennel1.Wagging_tail	Sociability	0.40	0.57	0.43	0.32	0.05	0.24
Kennel2.Wagging_tail	Sociability	0.16	0.63	0.20	0.24	0.09	0.17
Activity.JumpOnGlass	Anxiety	0.18	0.45	0.74	0.21	0.04	0.16
Kennel1.Licking_self	Anxiety	0.29	0.25	0.65	0.18	0.09	0.17
Activity.NearDoorFrame	Anxiety	0.29	0.43	0.78	0.34	0.03	0.22
BoldShy.ApproachCar	Curiosity	0.24	0.39	0.28	0.92	0.07	0.20
BoldShy.GazingAtCar	Curiosity	0.09	0.26	0.23	0.68	0.00	0.24
BoldShy.ProximityCar	Curiosity	0.26	0.44	0.29	0.88	0.05	0.19
BoldShy.RetreatCar	Curiosity	0.21	0.39	0.37	0.92	0.08	0.20
ExperimentDay	TimeInShelter	0.02	0.10	0.07	0.06	1.00	0.56
coughing	Illness	0.13	0.33	0.22	0.17	0.43	0.79
nose.condition	Illness	0.01	0.15	0.08	0.13	0.59	0.71
Temp	Illness	0.16	0.31	0.26	0.24	0.28	0.73

**Fig 4:**
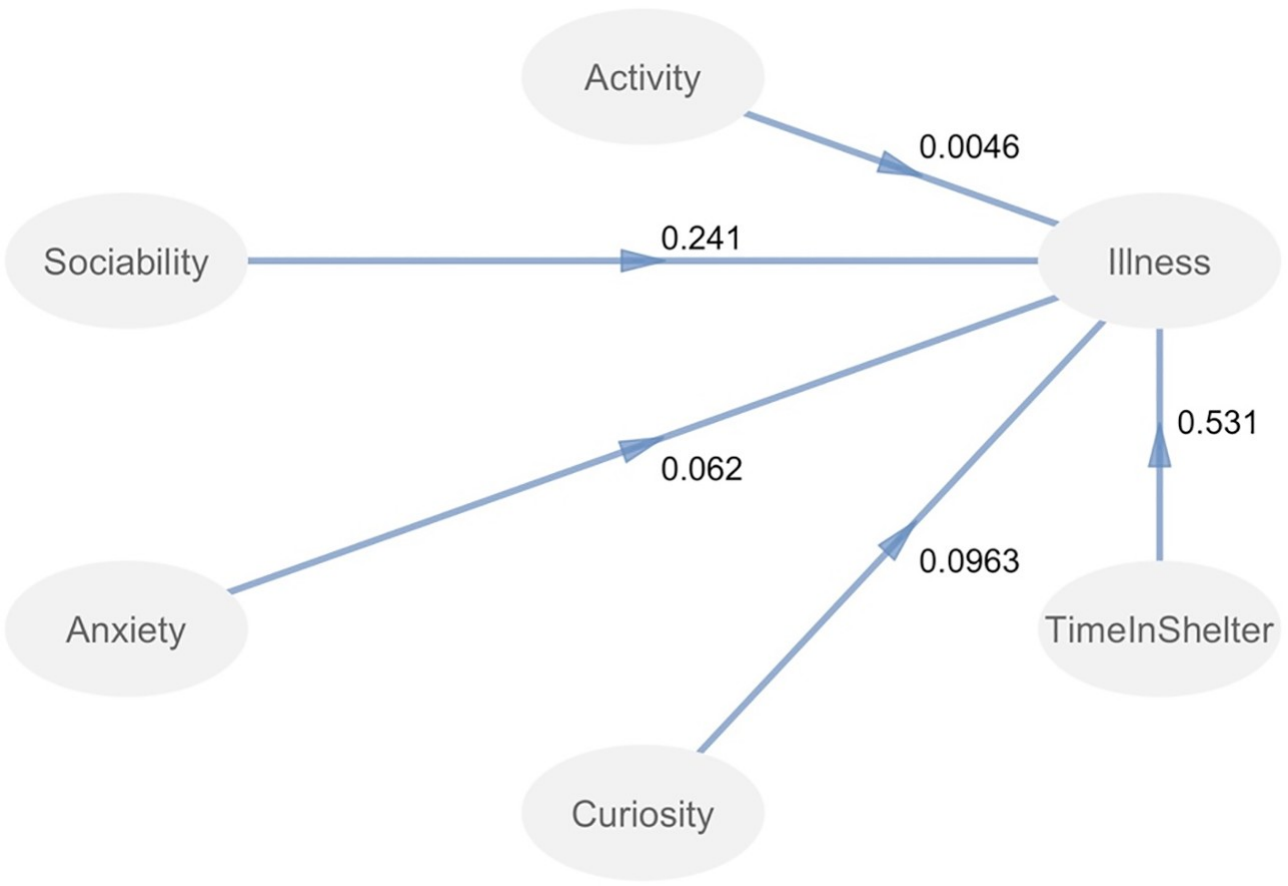
Path analysis model. The numbers are the proportion of variance explained with each variable out of the total variance explained by the model.

**Fig 5:**
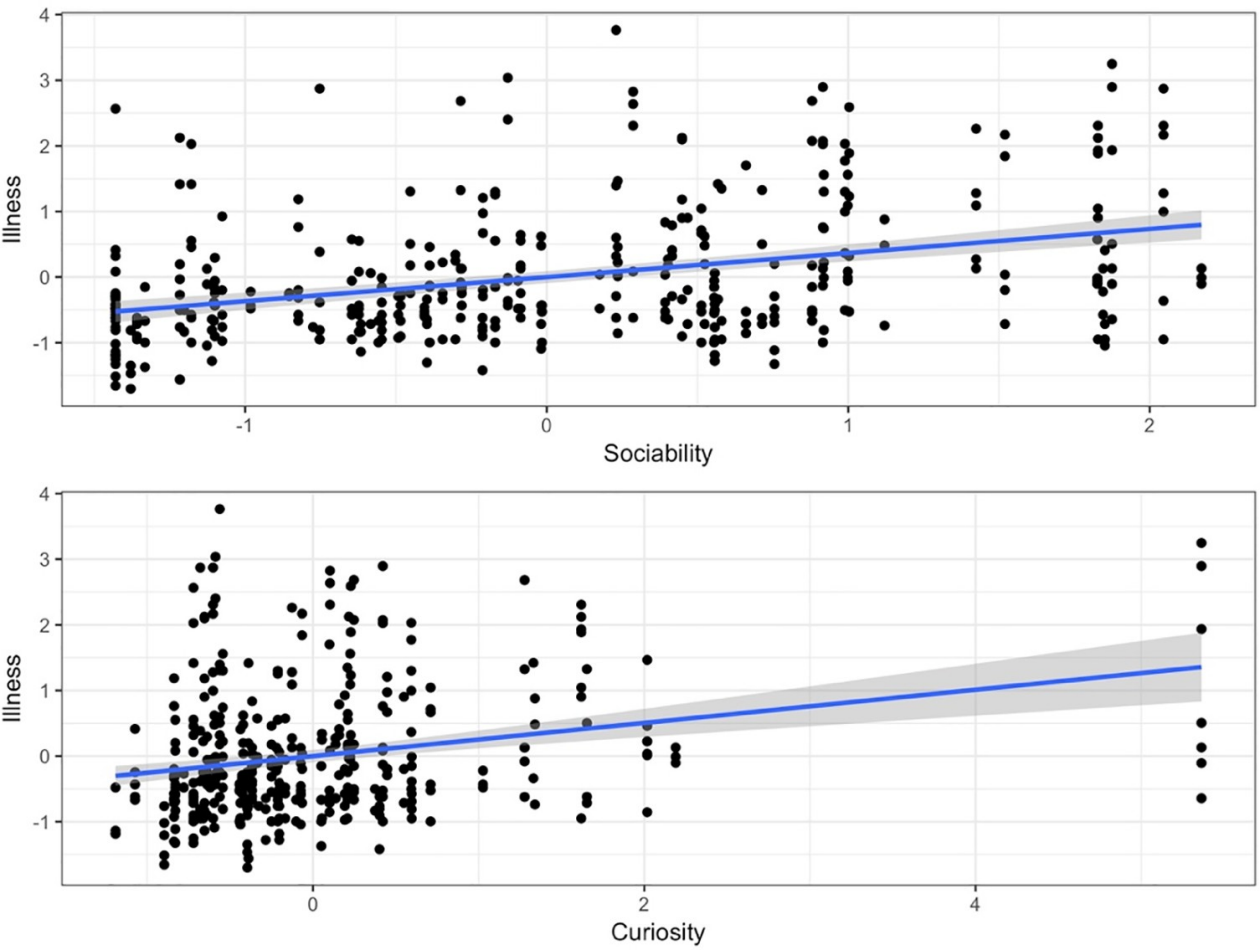
Effect of sociability and curiosity on illness scores. As Sociability and Curiosity increased, illness scores increased. Line shows linear regression and shading indicates model 95% confidence interval.

To evaluate whether Activity, Sociability, Anxiety, Curiosity, and Time in the shelter were related to the illness score, we conducted a basic PLS Path regression model in which our 5 latent variables were tested for association with illness. Fig 4 shows the hypothesized path model with regression coefficients. Table 9 shows the model estimates and statistical significance. Overall, Sociability, Curiosity and Time in the Shelter were significantly associated with illness. As expected, as time in the shelter increased, illness scores did also (estimate = .53, p < .001). Increases in Sociability scores (estimate = .24, p < .001; Fig 5) and Curiosity (estimate = .09, p = .026) were associated with increased illness. Activity and Anxiety, however, were not associated with illness.

**Table 9:** Path analysis model. Estimates, standard error (Std. Error), t value, p-value, and 95% Confidence Interval (CI) are shown for the model.

	Estimate	Std. Error	t value	p-value	95% CI (Boot strap estimate)
**Intercept**	9.20E-16	0.038	2.40E-14	1.00	
**Activity**	4.62E-03	0.042	1.10E-01	.91	-0.05–0.07
**Sociability**	2.41E-01	0.048	5.02E+00	< .0001*	0.156–0.34
**Anxiety**	6.20E-02	0.047	1.33E+00	.18	-0.01–0.14
**Curiosity**	9.63E-02	0.043	2.23E+00	.026*	0.01–0.16
**Time in Shelter**	5.31E-01	0.039	1.38E+01	< .0001*	0.44–0.59

### Urinary cortisol:Creatinine ratio

The mean C:C ratio was 18.4 x 10^−6^ (SD = 11.2 x 10^−6^). Due to missing C:C ratio data (19/83; 23% missing), C:C ratio data were excluded from the PLS path regression. To evaluate whether the C:C ratio was associated with illness, a linear mixed model with dog ID as a random effect and C:C ratio and time in the shelter as fixed effects indicated that the C:C ratio was not associated the illness score, although time in the shelter was (see Table 10).

**Table 10:** Linear mixed model with ID as a random effect, and cortisol and time in shelter as variables associated with illness score. Estimates, standard error (Std. Error), degrees of freedom (df), t value, and p-value are shown for the model.

	Estimate	Std. Error	df	t value	p-value
**(Intercept)**	-9.52E-04	7.77E-02	6.40E+01	-0.012	0.99
**TimeInShelter**	5.54E-01	4.23E-02	2.56E+02	13.106	<2e-16
**scale(C:C ratio)**	-3.63E-02	7.35E-02	6.96E+01	-0.494	0.623

To further explore in a reduced sample, whether the C:C ratio was associated with the latent behavioral variables, we computed a cross-correlation matrix between C:C ratio and our latent variable scores from our PLS Path model. C:C ratio was slightly negatively correlated with Sociability (r = -.22), indicating that more sociable dogs had lower C:C ratios. However, due to the large number of correlations and reduced sample size for this analysis, we did not compute p-value to interpret statistical significance. Lastly, the cross-correlations indicate Sociability and Curiosity were positively correlated (r = .42), suggesting these variables may be related and perhaps a more complex path analysis may be worth exploring in future studies with a larger sample size.

A correlation matrix was constructed with C:C ratio, time at the shelter, standardized illness, Sociability, and Curiosity scores. Time in the shelter and the standardized illness score had a moderate correlation of .52. Sociability and Curiosity scores had a moderate correlation of .42. Sociability had a moderate correlation with the Standardized illness score of .35. Standardized Curiosity and illness scores had a lower correlation of .20. C:C ratio had a lower negative correlation of -.23. No correlation was present between Sociability and time in the shelter, Curiosity and time in the shelter, and C:C ratio and illness score (Table 11).

**Table 11:** Correlation matrix correlation values.

	Sociability	Curiosity	Time	Cort:Creat	Illness
**Sociability**	1	0.415989	0.067201	-0.22718	0.354742
**Curiosity**	0.415989	1	-0.00806	-0.16363	0.196271
**Time**	0.067201	-0.00806	1	-0.04461	0.517382
**C:C ratio**	-0.22718	-0.16363	-0.04461	1	-0.07175
**Illness**	0.354742	0.196271	0.517382	-0.07175	1

## Discussion

In support of previous research, we found that time in the shelter was positively associated with the incidence of illness symptoms. Across time, each sign of upper respiratory illness (coughing, sneezing, ocular and nasal discharge, and fever) became more severe. Whereas increases in all signs of illness were already evident as early as the third day in the shelter, by two weeks, the average dog in this animal shelter had a fever, colored nasal discharge, clear ocular discharge, and half of dogs were coughing and/or sneezing. This data supports previous research that found that the risk of coughing increased by 3% each day[39].

Out of the four behavioral components, only Sociability and, to a much lesser extent, Curiosity, but not Activity or Anxiety was associated with illness. Dogs who had higher standardized scores in both Sociability and Curiosity in the first few days after intake, were more likely to have higher illness scores. Sociability consisted of tail wagging and jumping on the cage when a person came up to the kennel and jumping, leaning on, and staying in proximity to the person during the sociability test. Curiosity consisted of paying attention to the remote-controlled car (approaching, retreating from, gazing at, and staying in proximity to the car) during the boldness test. The decision to label these behaviors as “Curiosity” rather than “Boldness” was arbitrary and was informed by subjective opinion by the authors that the dogs’ behavior was more closely in line with the human concept of curiosity (i.e., information seeking) rather than boldness (e.g., a willingness to take risks). Furthermore, in our study, we did not assess for repeatability and thus are limited in the interpretations of our data in terms of personality or temperament literature.

According to visual analysis of the data, a clear positive linear relationship was evident between the standardized Sociability and Illness scores. However, the relationship appeared less clear between standardized Curiosity and Illness scores, with some potential outliers driving the positive correlation. It is also noteworthy that Sociability and Curiosity were moderately correlated, suggesting a potential underlying trait or that a more complex path model might be suggested for future larger studies. Previous research has suggested that the various behavioral components may be part of a greater whole. For example, Svartberg and Forkman[36] suggested that various traits, such as sociability and exploration, among others, may be related to a single higher-order dimension. Corsetti and colleagues[26] have also combined the individual traits of activity, attentiveness, dominance, and sociability to differentiate dogs into proactive and reactive coping styles. However, they found that dogs displaying the proactive style (higher boldness, higher sociability) had a lower incidence of illness; our current results seem to be contrary to this previous data. However, Corsetti et al.[26] did not find any statistically significant predictors when assessing individual traits, such as boldness, activity, sociability, or anxiety; the lack of statistically significant correlations among individual traits to illness may have been due to a relatively small sample size. Additional research may be needed to reconcile these contrary findings.

The values of urinary C:C ratios in our population (average of 18.4 x 10^−6^) were comparable to previous values (6.2 x 10^−6^[53], to 40 x 10^−6^[54]). Urinary C:C ratio, taken on the first full day, was not associated with subsequent illness in the animal shelter. Previous research suggests that coping style has a link with the responsiveness of the HPA axis. The proactive coping style has previously been found to correlate with low cortisol responses in dogs[40,41]. And in fact, we did find that cortisol had a low negative correlation with the sociability component. Interestingly, the same negative correlation of cortisol and sociability (but to conspecifics) was found in rhesus macaques[18]. However, instead of the proactive temperament protecting the dogs from illness (through the reduction of cortisol), we found a positive association between sociability and illness. As the correlation between cortisol and sociability was low, this finding may be a Type I error, and no true relationship may exist between the two in this population; alternatively, the relationship between proactive coping style and HPA reactivity may differ in this species or in this environment.

Our data may fit the Risk-of-Parasitism (RoP) hypothesis, which suggests that animals that exhibit bold or exploratory behavior encounter more parasites or pathogens (e.g.,[42]). The probability of encountering a parasite increases mechanically as the animal engages in exploratory behavior. For example, pumpkinseed sunfish that exhibited a bold temperament were more likely to have a higher parasite load[42]. Tom cats who exhibited a more dominant and bold temperament were also more likely to be infected with feline immunodeficiency virus[20]. Norway rats with higher testosterone were more likely to engage in fighting and more likely to have a hantavirus infection[21]. Wood mice that were infected by nematode exhibited more locomotion[43]. Similarly, in our study, dogs that were more curious and social may have encountered more infected surfaces, thus were more exposed to pathogens than dogs that were not curious nor social. However, during the time of the study, the animals were typically group housed in relatively small kennels with continuous rotation of animals and no sanitation prior to new arrivals. Therefore, the already very high risk of transmission in this particular shelter reduces the likelihood that the RoP hypothesis accounts for the entirety of these results. Nevertheless, this hypothesis remains a viable candidate for the explanation of the found phenomenon, and more data and experiments are required.

An additional hypothesis has been put forth to explain whole-animal differences in immune function, the Pace-of-Life-Syndrome (PoLS) hypothesis. The PoLS hypothesis has originally been used to differentiate different species by their “pace of life,” or metabolic and reproductive evolutionary strategy[44,45]. For example, some species may prioritize reproduction but not immune function or longevity. This strategy may be regarded as “live fast, die young.” In contrast, some species may prioritize longevity and immune function instead of reproduction—the “live slow, die old” strategy[45,46]. Recently, the PoLS hypothesis has been utilized to explain whole animal differences within a single species[47]. In fact, such differences have been previously suggested in dogs[48]. Thus, it is possible that intra-species differences in dogs may also follow these two evolutionary strategies, with one strategy prioritizing immune function and the other prioritizing reproduction. Perhaps dogs that are curious and social are utilizing the “live fast, die young” strategy, and are thus not prioritizing immune function. In fact, due to dogs’ reliance on human influence, perhaps human-directed sociability is a strategy for dogs to ensure medical care; thus, by putting more resources into sociability, fewer resources are needed for immune system function. Chersini, Hall, and Wynne[49] suggested that dogs may utilize human intervention for their survival; people rate pups at weaning as most desirable, and this is also the time when dams leave their pups to fend for themselves. With pup survival being only around 70% in free-ranging situations, human involvement becomes crucial to the dog[50]. While intriguing, our current data are not adequate for supporting or refuting this hypothesis. In order to provide support, future data need to show that social and curious dogs also have higher litter size, higher basal metabolic function, and shorter lives. In addition, future data would need to show that people will spend more money or effort on healthcare of social dogs.

According to the path analysis model, the outcome illness variable consisted of rectal temperature, the presence of nasal discharge, and coughing. Sneezing and ocular discharge did not load well onto the illness factor; however, it is noteworthy that both also increased with time, suggesting that they may be associated with the later part of disease progression, or with a distinct disease process.

Another important consideration is the potential paradoxical effect in which a positive correlation maybe observed across individuals but a negative correlation observed within individuals[51]. Thus, although we observed positive relationships with sociability and curiosity, the correlation at the individual level maybe negative, such that when a dog is more sociable than its typical average, it may be less likely to develop an illness. The present study, however, is limited in its ability to detect such effects due to our limit of only two data points from the first two observations per dog. Had we taken more longitudinal data for dogs that stayed for longer periods, this would have been an interesting analysis.

Several limitations were present in the current study. Due to human error, we were not able to assess the immune function of the dogs directly. Future research may need to verify the effects of temperament on immune function itself, rather than relying on the indirect measure of subsequent illness. However, in the shelter environment, the predictive nature of temperament remains to be meaningful, regardless of the underlying mechanism.

A second limitation was that 12 (14%) of the animals received vaccinations against pathogens that contribute to CIRDC, the target disease complex. Vaccination on intake can reduce disease incidence in shelters, probably through stimulation of innate immunity. Only a subset of these dogs (n = 9; 10%), however, received them during the data collection phase. Of the 9 dogs that were vaccinated during the data collection phase, 6 showed signs of illness post-vaccination (S1 Fig). It is likely that even the vaccinated dogs were still exposed to pathogens prior to vaccination. Nevertheless, future research may circumvent this issue by administering a vaccine challenge to all and measuring the immune response directly. This might also circumvent the problem of animals having unknown prior vaccination histories.

A third limitation was that dogs were continuously rotated through the kennels, thus resulting in different groups of dogs per kennel at the different observation times. This shelter procedure made it difficult to assess the Risk-of-Parasitism hypothesis as well as generally made it difficult to account for the effect of conspecifics during kenneling.

Finally, we also found high rates of attrition from the study. This limits the longitudinal sample size. To try to limit the effects of attrition, we focused our analysis on early prediction of illness and therefore utilized predictors obtained from the first three days of entering the study only, allowing us to have information on the predictors for all dogs. Further, our predictor of interest was health, and unfortunately, many dogs were becoming sick early on, with many dogs showing illness in the first week, while we still retained many of the dogs. Nonetheless, it’s unclear to what degree the data maybe censored due to euthanasia before developing an illness or going up for an adoption. Expanding on the current sample size would be an important follow-up study.

Regardless of the biological and/or evolutionary mechanism by which dogs with certain temperaments were more susceptible to illness, these data are important for the establishment of predictive models in the applied animal shelter environment. Providing knowledge about which dogs are more susceptible to illness, would allow shelter staff to manage the dog population more effectively. A suggestion may be to treat highly social animals as immunocompromised and manage these individuals similarly to nursing moms and puppies. Current best-practices suggest housing immunocompromised individuals in a different location away from the general population and taking additional care with disease transmission in these rooms[52]. However, further applied research is needed in order to develop behavioral screening assessments that would adequately predict subsequent illness.

## Conclusion

To summarize, we found a positive relationship between some temperament traits of dogs, namely Sociability and Curiosity, and subsequent signs of CIRDC illness in the animal shelter environment. Due to significant dropout of participants, however, we were not able to observe whether individual variability had a similar relationship. Further, we did not find any effect of urinary C:C ratio on subsequent illness. These data are contrary to previous pilot data that suggest that proactive temperaments may protect dogs from subsequent illness. Explanations for our data may include the Risk-of-Parasitism and Pace-of-Life Syndrome hypotheses. Future research is needed to differentiate between these two hypotheses as well as develop predictive models for use in animal sheltering.

## Supporting information

S1 File R file of raw data and analyses. The file contains all raw data and all analyses conducted in the statistical software R.
(RAR)

S1 Fig Individual illness scores of vaccinated dogs. Panel 1 shows the illness score of individual dogs that were vaccinated during the study period. The vertical line indicates the day in which the dogs were vaccinated. Panel 2 shows the comparison trend line for the remaining dogs as reference comparison.
(TIF)
