# Supplementary figures and images for: Behavioral predictors of subsequent respiratory illness signs in dogs admitted to an animal shelter
Source: PLoS One. 2019 Oct 23;14(10):e0224252. doi: 10.1371/journal.pone.0224252 (PMC6808433; doi:10.1371/journal.pone.0224252)

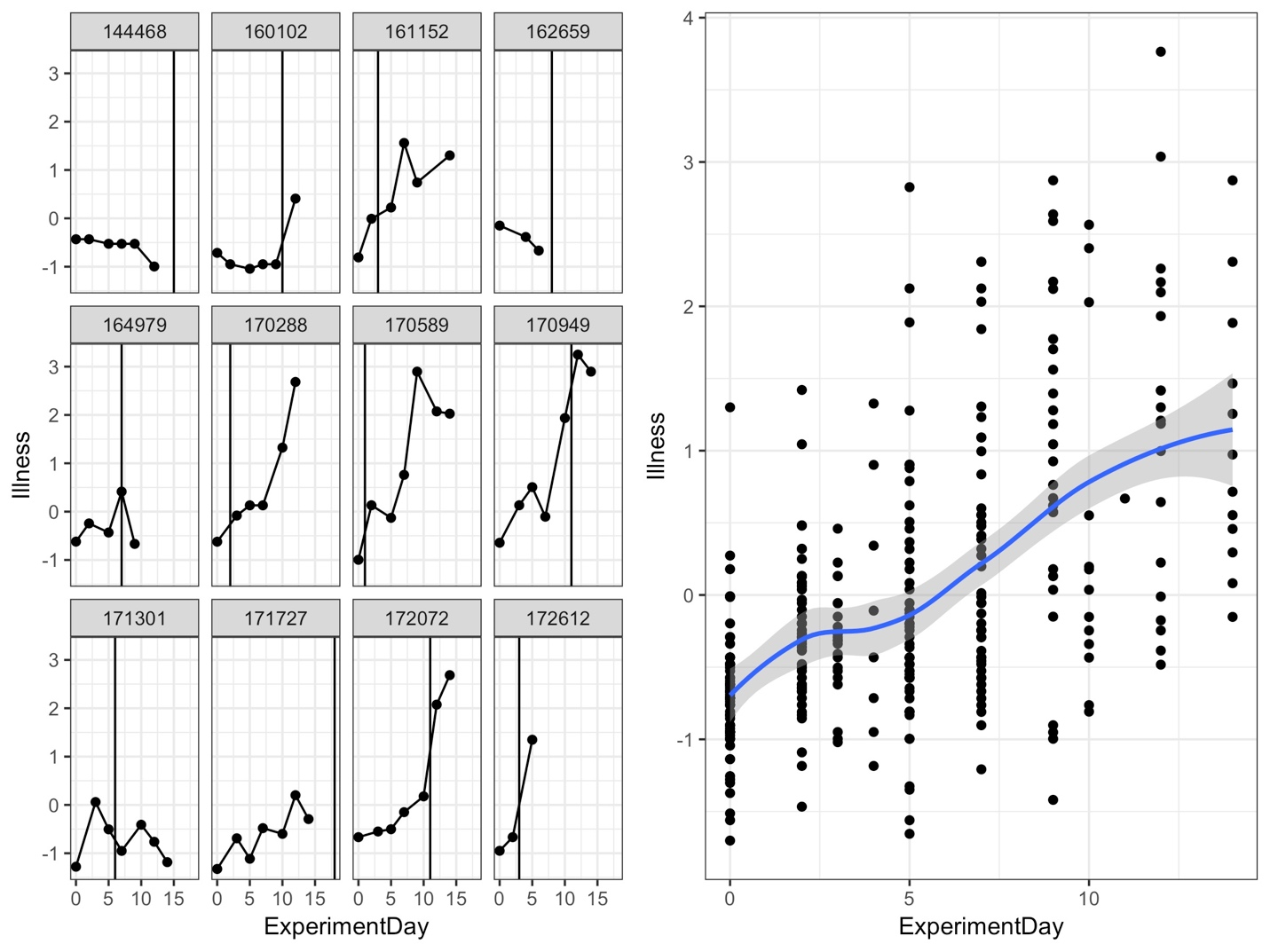

Supplement: S1 Fig — Panel 1 shows the illness score of individual dogs that were vaccinated during the study period. The vertical line indicates the day in which the dogs were vaccinated. Panel 2 shows the comparison trend line for the remaining dogs as reference comparison. (TIF) [file pone.0224252.s002.tif]
